# Neglected tropical diseases in the genomics era: re-evaluating the impact of new drugs and mass drug administration

**DOI:** 10.1186/s13059-016-0916-1

**Published:** 2016-03-14

**Authors:** Simon L. Croft

**Affiliations:** Faculty of Infectious and Tropical Diseases, London School of Hygiene & Tropical Medicine, Keppel Street, London, WC1E 7HT UK

## Abstract

Simon Croft answers *Genome Biology’s* questions on ways to approach neglected tropical diseases in the genomics era, including re-evaluating the impact of new drugs and mass drug administration.

## Could you provide a brief introduction to ‘neglected tropical diseases’? Why are they classified as ‘neglected’, which diseases are included in this category, and where do they mostly occur?

The concept of neglected tropical diseases (NTDs) was first proposed in the 1970s by the late Ken Warren. He brought attention to a large proportion of the human population who were poor, who suffered from chronic, disabling but rarely fatal diseases, and who were often stigmatized and unemployed. An excellent overview of Ken Warren’s insight and contribution has recently been published [1].

The World Health Organization (WHO) provides a list of 17 diseases [2]: protozoan parasitic diseases (leishmaniasis, Chagas disease, and human African trypanosomiasis), bacterial diseases (yaws, leprosy, Buruli ulcer, and trachoma), viral diseases (dengue, chikungunyu and rabies) and helminth diseases (schistosomiasis, cystercercosis or taeniasis, onchocerciasis, lymphatic filariasis, diseases caused by soil-transmitted helminths echinococcosis and dracunculiasis and food-borne trematodiases). Cases have been made for the addition of others (for example mycetoma [3]) and to remove some (for example dengue) that are subject to substantial impact and research investment in middle-income countries. The list of infectious diseases included as NTDs by major international organizations varies; some bodies distinguish NTDs from the ‘big three’—HIV/AIDs, tuberculosis and malaria—referring to them as 'diseases of poverty’ on the basis of funding availability [4].

Other than the label, the NTDs have little in common. They range from those with regional to those with worldwide distribution, from the potentially fatal to the disfiguring, from those that affect childhood growth to those that cause adult disease, from those that only effect people in poverty to those that also impact the affluent (for example, dengue and food-borne trematodiases). For some NTDs, tools for treatment and control are available; for example, there are vaccines for rabies and drugs for soil-transmitted helminths (STHs) and trachoma. For other NTDs, for example the trypanosomiases and leishmaniases, appropriate tools for treatment and control are still lacking.

The NTD label has had major success in raising the profile of this group of diseases over the past 15 years through a combination of advocacy and scientific and public health programs. This has ensured that NTDs are on the agenda of major international organizations, including the WHO, the UK's Department for International Development (DfID), and the Bill and Melinda Gates Foundation, all of which have dedicated NTD programs. In addition, organizations dedicated to NTDs, for example the Drugs for Neglected Diseases Initiative (DNDi, Geneva), have been established. In the mid-2000s, a time when there were but 13 NTDs listed, publications described the potential for disease control and the economic benefits of mobilizing resources for combined efforts around, for example, mass drug administration for the control of STHs and schistosomiasis (for example, [5, 6]). A sustained advocacy campaign culminated in the London Declaration [7] in which a coalition of international and philanthropic organizations, together with 14 pharmaceutical companies, committed to a program of donation and coordination to eliminate, eradicate or control ten NTDs by 2020. The impact of this commitment on policy has already been felt, and the success of initiatives will be evaluated over the coming years. But there remain several diseases for which sustained joint action by product development partnerships (PDPs), academia and the private sector is still required; these efforts are soon to be joined by antimicrobial resistance initiatives.

## Genomics research has revolutionized our understanding of infectious diseases, such as malaria, HIV and tuberculosis, and has provided some hope for tailored therapies through an improved understanding of host and vector genomics, and pathogen genetics. What impact, if any, has genomics had on our understanding of NTDs?

The genomes of most of the pathogens and some of the vectors responsible for NTDs have been described over the past 15 years, and this research has now become an integral part of most of our work on the target-based development of drugs, vaccines and diagnostics. The potential for genomics in drug discovery and development, well described over 14 years ago [8], has become increasingly sophisticated. Our understanding of genomics and molecular techniques has unraveled the mechanisms of action of anti-trypanosomal and anti-malarial drugs; an RNA interference (RNAi) approach was used for genetic validation in the former and conditional transcription expression systems in the latter [9]. Specific *Plasmodium falciparum* mutants, importantly those altered in the K13 propeller gene linked to artemisinin resistance, have provided a tool for chemogenomic profiling and the ability to map drug structures to targets [10]. Vaccine design [11] and re-design of diagnostics [12] have also benefitted from the integration of genomics tools. At the parasite population level, we now understand that the population structures of the pathogens that cause visceral leishmaniasis and schistosomiasis are linked to resistance to pentavalent antimonial drugs [13] and to praziquantel [14].

One area that deserves further exploration to ensure that we design and use drugs and vaccines for NTDs effectively is pathogen diversity, exemplified in recent genomic studies on the pathogens *Plasmodium falciparum* and *Mycobacterium tuberculosis* [15, 16]. Studies on vaccines, RTS/S/ASO1 for malaria and BCG for tuberculosis, have shown the importance of knowing the genetic similarity of the circulating pathogens to the model strains from which the vaccine is constructed. The efficacy of the malaria vaccine is related to allele-specific protection associated with the NANP–NVDP epitope of the polymorphic circumsporozoite protein, and is linked to the proportion of matched alleles in local *P. falciparum* populations [17]. In a similar vein, variation in BCG effectiveness could result from significant genetic variation in the strains used in immunization [18]. To ensure that we design the most effective and appropriate tools for NTDs, lessons must be learnt and account taken of genetic differences in pathogen populations in different regions; this concern was built into our design of a vaccine for leishmaniasis [11].

At the same time as host-directed drug therapy is gaining importance in anti-infective discovery [19, 20], advances in human genomics have begun to play a role. Genome-wide association studies (GWAS) have identified infectious disease susceptibility factors, for example to dengue and leprosy [21], and exploitation of this knowledge in relation to immune response pathways is underway. In drug development, the importance of pharmacokinetics in different populations has long been recognized, and pharmacogenomics is recognized as being a key factor in the discovery of novel anti-infectives [8]. More recent work has described the significant polymorphism of key drug metabolizing Cyp genes in Africa and in other regions [22].

## The ‘World Intellectual Property Organization’ has previously summarized the attitude to drug development for neglected tropical diseases as ‘market failure’, or non-profitable. How does this attitude relate to the paucity of genomics studies on these diseases?

For all infectious diseases, drug development is a complex process rather than the linear process often portrayed [23]. The role of genomics is often seen as being in the early part of drug discovery, related to the identification of novel drug targets; but much of the understanding of pathogens derived from genomics has been or could be used in other ways. Over the past five years, two significant changes have improved drug R&D. First, the remarkable shift in the approach of the pharmaceutical industry to those wishing to access and use their drug libraries, often with publication of data. This has been a catalyst for several new projects and partnerships between sectors. The second, and linked, advance has been in the application of phenotypic high throughput (HTS) and high content screening (HCS) technologies and their integration into the drug discovery pathway, earlier described for NTDs by Frearson et al. [24]. The potential of this approach has been illustrated by the publication on anti-malarial compounds from the GSK screen [25], and more recently for the NTDs by the publication of compounds active against *Trypanosoma* and *Leishmania* parasites [26].

So how does all this relate to market failure ('a situation in which the allocation of goods and services is not efficient' *Wikipedia*)? This has been a major concern for the WHO who, through mechanisms such as the Working Group on Research and Development: Finance and Coordination, have been discussing new 'global instruments' for research funding for the past decade. For the diseases of poverty (HIV, malaria, and tuberculosis), the Global Fund (www.theglobalfund.org) invests in endemic countries to mobilize and support the cost of purchasing of many drugs. This also helps the companies producing the drugs and vaccines, for whom the concept of market is essential as it helps to predict demand and scale of manufacture. An expansion of the Global Fund remit to include NTDs could have a major impact. This has been done elsewhere; the European & Developing Countries Clinical Trials Partnership (EDCTP), which supported clinical studies on ‘the big three’, expanded its remit in 2014 to include NTDs. There are also other vehicles to encourage engagement with this market, ranging from tax credits to voucher schemes, the latter are already in action [27], although issues have been raised over their use for NTD tool innovation [28]. What is also needed for NTDs is: (i) improved and accurate data on disease in many endemic countries; and (ii) greater engagement of the endemic countries in the R&D process from bench to bedside. There are promising signs for the latter point with the potential of the first anti-malarial drug to be developed in Africa (http://h3d.co.za). Although this might seem a long way from genomics, a clearer understanding of what tools are needed and how they will be used should help to engage basic scientists in the process, for example through engagement with target product profiles [24, 29].

## Only 10 % of global research and development resources were allocated for neglected diseases between 1975 and 2000, and only 13 new drugs were approved for their treatment within this time period. What gaps are there in our understanding of NTDs?

A recent report has described a small improvement in the number of drugs approved from 1.1 % in 1975–2000 to 4 % of drugs approved over the period 2000–2011 [30], although also highlighting the remaining gaps for drugs and vaccines. The limited progress for NTDs has to be placed in context. The lack of new drugs and vaccines since the 1980s, with some exceptions, applies equally to the discovery and development of new antimicrobial drugs in general [31, 32]. The current alarm over antimicrobial resistance has pointed to the need for both additional investment in antibacterial drugs and the commitment of pharmaceutical companies to this area of research, issues raised by the international health community [33] and by governments [34]. Only two new drugs for tuberculosis have been developed in the past 30 years [35]. There is no difference in the R&D process for NTDs and other infectious diseases: the same skill sets and the same pathway from bench to clinic are required. Some encouraging developments over the past decade show a way forward: (i) industry, public–private partnerships and academia have engaged and, for some diseases such as malaria, there is now a healthy portfolio of new products [36]; (ii) re-analysis has shown why some anti-infective drug discovery campaigns have failed [31, 32]; and (iii) there is a recognition of how disease models must be improved to facilitate the R&D process for NTDs [37, 38]. The recent successful development of a series of new drugs and drug combinations for the hepatitis C virus shows that when knowledge, effort, skills, facilities and investment are combined across academia and industry, many issues can be resolved rapidly [39].

## Could you provide a brief introduction to mass drug administration for the treatment of neglected tropical diseases? How does it work, and for which diseases is it used?

Mass drug administration (MDA) is used on whole populations, irrespective of individuals' disease status, to control, prevent or eliminate common or widespread disease. It has been widely used for infectious diseases in the past, for example for malaria in the 1950s [40], and continues to be used widely in veterinary medicine to prevent helminth infections in cattle and sheep. With regard to NTDs, MDA normally refers to use for helminth infections namely STHs, the filariases and schistosomiasis, with albendazole and ivermectin being used for the former two and praziquantel for the latter and to the use of azithromycin to prevent bacterial infection trachoma. MDA has been advocated for helminth diseases over the past decade [41], with delivery to affected populations on an annual or bi-annual basis, often integrated into other infectious disease programs. MDA has also recently been reconsidered for prevention of malaria, including attempts to control of the spread of artemisinin resistance [42]. The more sophisticated approach of mass screening and treatment (MSAT), whereby drugs are administered only to individuals who test positive for the pathogen, has advantages. Whereas both MDA and MSAT rely upon high coverage and repeated interventions to achieve a long-term effect, MSAT also requires the availability of adapted and appropriate diagnostic tools. MSAT has been applied to malaria [43] and could well be used for the other NTDs such as human trypanosomiasis in sub-Saharan Africa and leishmaniasis in the Indian sub-continent.

## The continued use of mass drug administration is controversial in the genomics era given all that we know about host, vector and intermediate host genomics, and pathogen genetics. Please could you explain why mass drug administration is controversial, and why it is still in widespread use?

MDA does raise important issues for consideration. First, as drugs are being administered to a whole population, which includes infants and women of child-bearing age who do not have the target disease, safety becomes a major consideration. The need for effective pharmacovigilance (the collection of data to allow the detection, assessment, monitoring, and prevention of the adverse effects of pharmaceutical products) is being adopted by many countries. In relation to NTDs, the WHO Collaborating Centre for International Drug Monitoring, Uppsala, established in 2009 the WHO Collaborating Centre for Advocacy and Training in Pharmacovigilance, Accra, Ghana [44], with a network of 40 countries in Africa.

Second, although the number of pills or doses donated to MDA campaigns for NTDs is in the millions, there are critical issues around both the ability of health systems to deliver the drugs to target populations and the compliance/adherence of populations in taking the drugs appropriately [45]. The impacts of these issues were detailed in relation to malaria eradication by the MalEra team [46].

Third, there is also a need to ensure the political and economic will to sustain MDA campaigns, especially when the number of cases decreases. A crucial decision is to know when 'enough is enough' and when the elimination or eradication goals have been achieved, and whether the target numbers are valid in relation to the prevention of disease transmission [47]. The determination of the 'endpoint' is being seriously considered, for example for lymphatic filariasis with carefully designed transmission assessment surveys [48]. One issue, linked to this, is to ensure that the criteria are fully validated. For example, the impact of MDA de-worming campaigns on attendance and educational performance in schools in Africa and other regions has been recently questioned following a re-analysis of the original data of a study in Kenya that found no effect on school examination performance [49].

Fourth, there is concern about MDA leading to drug and insecticide resistance, which is frequently raised and based on long experience in the veterinary field. The importance of genomics in identifying drug and insecticide targets is an established part of research in this area. Research on specific insecticide and anti-parasitic target genes of agents used in control programs highlights the importance of this approach [50, 51]. Nevertheless, a review on MDA for schistosomiasis shows how genomics and genetics can be used to provide an understanding of population structure, transmission, potential spread of drug resistance and models that can be used to evaluate the effect of MDA and to determine cut-off points; this is an excellent example of the integration of genomics into disease control [14].

There are other considerations. In relation to STH, Vercruysse et al. [52] concluded that there was limited evidence for the development of resistance, but drew attention to variation in drug effectiveness resulting from significant differences in the efficacy of albendazole and mebendazole against different helminths, the impact of seasonal transmission and the absence of a surveillance system. For some diseases included in MDA programs, for example schistosomiasis, there is also a pressing need to consider what actions to take if resistance to praziquantel does occur; it is unlikely that the new wave of drug screening against *Schistosoma* [53, 54] will lead to the development of a new drug in time.

## Can genomics lead to the development of alternative treatments and control methods for NTDs?

Some of the areas where genomic analysis can contribute to drug development are referred to above. There are other areas where understanding of genomes and molecular biology could lead to new therapies. The recently much-vaunted CRISPR technology has been used to investigate the biology of bacteria and protozoa. The CRISPR-Cas systems have already been considered as a route to new antimicrobials [55], with CRISPR-Cas9 antimicrobials able to kill *Staphylococcus aureus* in vivo in a mouse skin colonization model [56]. Antisense oligonucleotide drugs were making headlines 20 years ago, and one (fomivirsen) was approved by the FDA in 1998 as a drug for cytomegalovirus infection. Research on the anti-parasitic effects of anti-sense oligonucleotides continues, for example in Chagas disease [57], but these compounds hardly fit the target product profile of drugs (oral, short course) required to treat NTDs. The potential that genomics will lead to novel approaches for vaccine development is greater (see [58] for a recent review with a particular focus on malaria).

## What is next for your work in this area?

One of the most neglected diseases is cutaneous leishmaniasis (CL), a parasitic disease that rarely kills but can have a devastating impact on individuals, causing disfigurement and stigmatization. For CL, treatment is limited because the drugs that are used have known toxicity, they normally require injection and have variable efficacy depending on the species of *Leishmania* involved [59]. Recent Cochrane reviews have highlighted the paucity of data coming from clinical trials on CL, many of which lack proper trial design [60]. For CL, there is an absence of clear strategy and leadership for drug R&D. I am currently working with DNDi on the evaluation of novel compounds for this disease, and also pursuing a long-held goal of rational design of topical treatments for CL, working closely with pharmaceutical and skin experts [61, 62].

## Abbreviations

CL: cutaneous leishmaniasis
DNDi: Drugs for Neglected Diseases Initiative
MDA: mass drug administration
MSAT: mass screening and treatment
NTD: neglected tropical disease
STH: soil-transmitted helminth
WHO: World Health Organization
